# Implications for optimizing treatment timing: day of week variation in PTSD symptom clusters

**DOI:** 10.3389/fpsyt.2025.1599424

**Published:** 2025-10-17

**Authors:** Quinn M. Biggs, Jing Wang, Carol S. Fullerton, Rohul Amin, Joseph A. Hooke, Natasha Dhanraj, Robert J. Ursano

**Affiliations:** ^1^ Center for the Study of Traumatic Stress, Department of Psychiatry, Uniformed Services University of the Health Sciences, Bethesda, MD, United States; ^2^ Henry M. Jackson Foundation for the Advancement of Military Medicine, Inc., Bethesda, MD, United States; ^3^ Walter Reed National Military Medical Center, Bethesda, MD, United States

**Keywords:** posttraumatic stress disorder, symptom clusters, ecological momentary assessment, treatment timing, military personnel

## Abstract

**Introduction:**

Posttraumatic stress disorder (PTSD) has four symptom clusters: intrusion, avoidance, negative cognitions/mood, and hyperarousal. Little is known about day-to-day variation in the symptom clusters. If there is such variation, it highlights the need to develop more person-specific interventions. This study examined the day of the week and weekday versus weekend variation in PTSD symptom clusters in individuals with and without PTSD.

**Methods:**

Using an ecological momentary assessment methodology, participants (*N* = 159; 80 with probable PTSD, 79 without PTSD) completed self-report assessments of posttraumatic stress symptoms (PTSS) four times daily for 15 days. Linear mixed models were used to examine associations between the seven days of the week and weekday versus weekend variation in PTSD symptom clusters and PTSD.

**Results:**

All four symptom clusters varied across the seven days of the week among participants with PTSD (intrusion: *p* = .038, avoidance: *p* <.001, negative cognitions/mood: *p* = .007, hyperarousal: *p* <.001), but not among those without PTSD. Among those with PTSD, the four symptom clusters were higher on weekdays than weekends (intrusion: *p* = .008, avoidance: *p* = .002, negative cognitions/mood: *p* <.001, and hyperarousal: *p* <.001). However, among those without PTSD, weekdays were higher than weekends only for the intrusion (*p* = .042) and hyperarousal (*p* = .017) clusters. Differences in when symptom clusters peaked were also noted.

**Conclusions:**

Among individuals with PTSD, the four symptom clusters vary by the day of the week with more symptoms on weekdays compared to weekends. Identification of the factors associated with day-to-day variation in PTSD symptom clusters may be important for understanding the neurobiology of PTSD and for treatment.

## 1 Introduction

Posttraumatic stress disorder (PTSD) is associated with multiple negative outcomes including detrimental effects on mental and physical health and quality of life (1, 2). In the general U.S. population, PTSD has an estimated lifetime prevalence of 6.4-6.8% (3, 4) and a 12-month prevalence of 3.5% (5). Among combat veterans, the prevalence is higher with the most commonly cited estimate of current PTSD being 15% (6, 7). The Diagnostic and Statistical Manual of Mental Disorders-Fifth Edition (DSM-5) identifies 20 posttraumatic stress symptoms (PTSS), which are grouped into four symptom clusters: intrusion, avoidance, negative cognitions/mood, and hyperarousal (8).

Examining individual aspects of a disorder may inform both our understanding of the underlying mechanisms and our approaches to care for the disorder. PTSS do not remain static. Studies have shown that PTSS fluctuate across time (9) and, specifically, the PTSS sum score varies across the days of the week and differs between weekdays and weekends (10). However, we do not know the extent to which the four DSM-5 PTSD symptom clusters vary from day-to-day. The presence of daily variation in PTSD symptom clusters may reflect patterns in neurobiological, environmental, psychosocial, or lifestyle factors. PTSD is thought to involve dysregulation of normal fear processes and the neural circuitry underlying fear and threat-related behavior (11). Little is known about the neurobiology of PTSD symptom clusters, although research has identified PTSD-related behaviors associated with specific neural circuits (12, 13). Better understanding the variation in PTSD symptom clusters may lead to new insights into the neurobiology of PTSD and development of more tailored treatment planning (14).

Prior studies suggest that the symptom clusters may differ in their contribution to the development and maintenance of PTSD (15). Hyperarousal has been shown to predict PTSD severity and changes in other symptom clusters (16–18). For example, when PTSD symptom clusters were assessed every 2 weeks for up to 2 years, hyperarousal predicted subsequent fluctuations in the three other clusters at subsequent 2-week intervals (17). However, most prior studies examining PTSS have used retrospective assessments, which ask respondents to recall symptoms over the past month. Retrospective assessments are subject to recall bias and do not capture potential moment-to-moment symptom variation nor the temporal relationships between symptoms that may be important for understanding PTSD (19, 20). In contrast, ecological momentary assessment (EMA) approaches, which involve repeated real-time sampling of respondent experiences in their natural environment, minimize recall bias, maximize ecological validity, and allow for the examination of contextual influences on behavior and temporal relationships of interest (21–23).

The purpose of the present study is to explore whether PTSD symptom clusters (intrusion, avoidance, negative cognitions/mood, hyperarousal) vary by the day of the week, and whether there is a relationship between symptom clusters and the day of the week. We used an EMA methodology to determine the day-to-day and weekday versus weekend difference in PTSD symptom clusters in U.S. military Service Members with and without PTSD during their typical daily routines. PTSS were assessed four times a day for 15 consecutive days, which allowed us to examine the temporal patterns of PTSD symptom clusters and the extent to which symptom clusters differ between weekdays, when most individuals are in their work environments and work demands are present, and weekends, when the demands of the work week are usually lessened. We hypothesized that because daily work demands would be higher and being in a military work environment may increase exposure to trauma reminders that may trigger symptoms, PTSD symptom cluster scores would be higher on weekdays versus weekends in those with PTSD but not among those without PTSD. We elected to include those without PTSD as their symptom scores may reflect the daily variation in stress symptoms associated with normal life stressors. To our knowledge, this is the first study to examine the day of the week variation in PTSD symptom clusters.

## 2 Methods

### 2.1 Participants

A total of *N* = 309 current and former U.S. Service members were screened for eligibility to enroll; *n* = 219 screened in. Of those, *n* = 183 enrolled, *n* = 8 declined enrollment, and *n* = 28 did not return for the enrollment appointment. Of the *N* = 183 who enrolled, *n* = 159 were included in data analyses, *n* = 14 did not return the momentary assessments, *n* = 8 did not provide three or more momentary assessments, *n* = 1 did not complete the assessment of PTSD in the baseline questionnaire, and *n* = 1 was removed as an outlier. This study was part of a larger data collection project examining posttraumatic stress in U.S. Service members. Prior publications (10, 24, 25) report the methods common to the larger project (e.g., recruitment and screening). The Institutional Review Boards of Walter Reed National Military Medical Center and the Uniformed Services University of the Health Sciences in Bethesda, Maryland, approved the study. We explained study procedures prior to obtaining written informed consent. Participation was voluntary.

### 2.2 Procedure and measures

#### 2.2.1 Recruitment and enrollment screening

Recruitment was conducted at a military treatment facility. Service members self-referred from advertisements or approach to a recruiting table. Participation in the study was independent of medical records and any mental or medical healthcare the Service member may have been receiving. Inclusion criteria included being 18 years of age or older, fluent in English, and from a uniformed service (Air Force, Army, Army National Guard, Army Reserves, Coast Guard, Marine Corps, Navy, and U.S. Public Health Service). Exclusion criteria included suicidal or homicidal behavior in the past three months or diagnosis of or care for a psychotic disorder in the past year. Service members meeting these criteria completed a 26-item screening questionnaire (10).

#### 2.2.2 Demographics

After enrollment, participants completed a baseline assessment, which included demographic characteristics: gender, age, race (White versus Non-White), and education level (some college or lower versus bachelor’s or higher). Participants also reported their Service affiliation (e.g., Army), current status (e.g., active, inactive, veteran), and rank (e.g., E-5).

#### 2.2.3 Assessment of probable PTSD

To determine probable PTSD status, participants completed an assessment of exposure to traumatic events and the 20-item Posttraumatic Stress Disorder Checklist for DSM-5 (PCL-5) (26) at baseline. A probable PTSD diagnosis required one or more cluster A traumatic exposures (all participants had at least one qualifying exposure), one or more items from clusters B and C, two or more items from clusters D and E, and a symptom severity score of >38 (26). Prior studies have used both DSM criteria and a cutoff score to determine PTSD in military samples (27). A cutoff of 38 is recommended for accurately measuring population prevalence consistent with previous military studies (27–29). See a prior publication (24) for details of the traumatic exposure measures and criteria for probable PTSD. The PCL-5 is a psychometrically sound instrument with good internal consistency (α = .96), test–retest reliability (*r* = .84), and convergent and discriminant validity; it is well-suited for assessing probable PTSD diagnostic status and symptom severity in current and former Service members (30, 31). Of the *N* = 159 participants, *n* = 80 met criteria for probable PTSD (hereafter referred to as those with PTSD) and *n* = 79 did not meet criteria for PTSD.

#### 2.2.4 Assessment of probable major depressive disorder

To determine probable major depressive disorder status at baseline, participants completed the Patient Health Questionnaire Depression Scale (PHQ-9) (32, 33), which is a valid screen for probable depression in military populations (34). Summed responses yielded a symptom severity score (range 0-27). A probable major depressive disorder diagnosis required >5 symptoms rated *More than half the days* or higher and one symptom was depressed mood or anhedonia.

#### 2.2.5 Momentary assessments

In the 15 days following enrollment, participants completed four momentary assessments per day using an EMA methodology (10). Assessments were set at fixed intervals, four hours apart, to capture symptoms across most waking hours similar to time block designs in other studies (35). Participants selected the hour of their first momentary assessment that best fit their daily routine (i.e., if the participant elected to start the first assessment at 8 a.m., then the second, third, and fourth assessments were at 12 p.m., 4 p.m., and 8 p.m., respectively, and these times remained consistent throughout the 15 days). We instructed participants to complete assessments within the first two hours, when possible, although the assessment window remained open for an additional four hours. Assessments completed before the assessment window began (too early), after it concluded (too late), or missing the completion date or time were invalid. The 15-day duration provided a buffer for potential missing data and yielded two full weeks of assessments per participant to support weekday/weekend comparisons. To diminish the potential for a decline in assessment compliance (36, 37), we contacted participants approximately every 3 days to reinforce study engagement and address any questions or problems. We collected *N* = 7,761 of the 9,540 possible assessments during the acquisition period. Completion rates across the 15 days ranged from 68% to 92%, with the following day-by-day rates: 89%, 92%, 86%, 83%, 85%, 79%, 79%, 80%, 75%, 76%, 73%, 76%, 75%, 71%, and 68%. Age was positively associated with completion rate, such that older participants tended to complete more assessments. We observed no significant differences in completion by gender or PTSD group. The pattern of missing assessments associated with study day and age was consistent with previous research (e.g., 36, 37). Of the collected assessments, *N* = 7,591 assessments were included in the data analyses (*n* = 170 [2.2%] were dropped because they were completed too early [*n* = 41], too late [*n* = 47], were missing the completion date or time [*n* = 81], or due to an error in electronic data [*n* = 1]). The overall assessment adherence rate was 79.6%. Of the *N* = 7,591 assessments included in the analyses, participants completed *n* = 5,776 (76.1%) within 0–2 hours, *n* = 1,313 (17.3%) within 2–4 hours, and *n* = 502 (6.6%) within 4–6 hours of the 6-hour assessment completion window. Participants were not compensated for completing assessments.

#### 2.2.6 PTSD symptom clusters

Daily PTSS were assessed using 18 non-sleep PCL-5 items (26), which were included on all four momentary assessments (see **Supplementary Figure S1**). The PCL-5’s two sleep-related items (i.e., item 2. *Repeated, disturbing dreams of the stressful experience* and item 20. *Trouble falling or staying asleep*) were not appropriate for use on all four assessments. We modified the response format of the PCL-5 items to an 11-point scale, 0 (*Not at all*) to 10 (*Extremely*). Prior research suggests that changing a 5-point scale to an 11-point scale produces data with more variance (38). We also modified the instructions to be relevant to the timing of the assessments; instructions in the first assessment contained the timing phrase “…*since you awakened*” and instructions in the second, third, and fourth assessment contained the phrase “…*in the last couple of hours*.” The validity of momentary assessments of PTSD symptoms using PCL-5 items has not been fully established. However, prior studies have repeatedly demonstrated their robust correlations with past-month estimates, which strongly support their construct validity (9, 39, 40). The correlation between the full 20-item PCL-5 administered at baseline and the person-level mean of the 18 non-sleep PCL-5 items in momentary assessments was *r* = 0.72, indicating strong convergence between the two measures. Also, we assessed internal consistency for each PTSD symptom cluster for the first available assessment completed by each participant. Cronbach’s alpha was 0.95 for intrusion, 0.86 for avoidance (calculated as a correlation due to there being only two items), 0.92 for negative cognitions/mood, and 0.82 for hyperarousal. To facilitate clearer visual and interpretive comparisons across clusters, we calculated item means for each of the four PTSD symptom clusters with a range of 0-10.

#### 2.2.7 Day of week and weekday/weekend variables

To test if the outcome differed by the day of the week, a 7-day and a dichotomous weekday (Monday through Friday)/weekend (Saturday and Sunday) variable were created. We did not include a general variable for time in the analyses because we did not find a developmental trend by time in the PTSD symptoms clusters.

### 2.3 Data analyses

Linear mixed models were used to examine the day of week variation in the four PTSD symptom clusters (intrusion, avoidance, negative cognitions/mood, hyperarousal), with momentary assessments (level-1) nested within subjects (level-2). Separate models were conducted for each symptom cluster. To account for unequal time intervals, a spatial power covariance structure was specified. The first-order autoregression assumption, AR (1), was used to model serial autocorrelation, as it provided better model fit compared to compound symmetry. This structure assumes that the correlation between two assessments decreases exponentially as the time interval between them increases. Three covariance parameters are reported: between-person variance (i.e., variance in average levels across individuals), the correlation between residuals of observations taken 4-hour apart, and within-person variance (i.e., residual variance representing variation in repeated measures within individuals across time). For each PTSD symptom cluster, we first estimated an unconditional (empty) model to assess the proportion of variance attributable to between-person differences, and we reported the intraclass correlation coefficient (ICC). We examined both the seven days of the week and the differences between weekdays and weekends. To assess whether results were robust to individual variability, we compared estimates from models that included versus excluded a random slope for weekday and weekend differences.

Since we were particularly interested in examining the day of week variation among participants with and without PTSD, we conducted stratified analyses by PTSD group. We included gender, age, race, and education as covariates. We used the Tukey-Kramer method to adjust for multiple pairwise comparisons. All analyses were performed using PROC MIXED in PC SAS version 9.3 (SAS Institute, Cary, North Carolina).

## 3 Results

### 3.1 Sample characteristics

Sample characteristics and descriptive statistics are shown in **Table 1**. Mean age of participants in the analytic sample (*n* = 159) was 41.5 (range 19-69). The majority (59.8%, *n* = 95) were male, White (64.8%, *n* = 103), had a bachelors or graduate degree or higher (55.3%, *n* = 35), and were married (65.4%, *n* = 104). Most were in the Army (40.3%, *n* = 64) or Navy (40.3%, *n* = 64), active-duty (62.9%, *n* = 78), and enlisted (59.4%, *n* = 89), and 31.0% (*n* = 48) had major depressive disorder. In total, 80 (50.3%) had PTSD and 79 (49.7%) did not have PTSD. Except for major depressive disorder, there was no difference between those with PTSD and without PTSD in demographic characteristics.

**Table 1:** Demographic characteristics and descriptive statistics of participants with and without PTSD.

Demographic Characteristic	Total (*N* = 159)	PTSD (*n* = 80)	Without PTSD (*n* = 79)
Categorical	*N* (%)	*N* (%)	*N* (%)
Gender			
Male	95 (59.8)	44 (55.0)	51 (64.6)
Female	64 (40.3)	36 (45.0)	28 (35.4)
Race			
White	103 (64.8)	52 (65.0)	51 (64.6)
Non-white	56 (35.2)	28 (35.0)	28 (35.4)
Education			
High school or GED	7 (4.4)	5 (6.3)	2 (2.5)
Some college/technical school	64 (40.3)	34 (42.5)	30 (38.0)
Bachelor’s degree	29 (18.2)	16 (20.0)	13 (16.5)
Graduate degree	59 (37.1)	25 (31.3)	34 (43.0)
Marital status			
Currently married	104 (65.4)	51 (63.8)	53 (67.1)
Not currently married	55 (34.6)	29 (36.3)	26 (32.9)
Living with spouse (among married)			
Yes	82 (76.6)	38 (71.7)	44 (81.5)
No	25 (23.4)	15 (28.3)	10 (18.5)
Service			
Air Force	17 (10.7)	7 (8.8)	10 (12.7)
Army	64 (40.3)	30 (37.5)	34 (43.0)
Navy	64 (40.3)	35 (43.8)	29 (36.7)
Other	14 (8.8)	8 (10.0)	6 (7.6)
Current status			
Active	78 (62.9)	40 (64.5)	38 (61.3)
Inactive	2 (1.6)	1 (1.6)	1 (1.6)
Veteran	44 (35.5)	21 (33.9)	23 (37.1)
Rank			
E2-E4	31 (20.7)	14 (18.4)	17 (23.0)
E5-E8	58 (38.7)	37 (48.7)	21 (28.4)
O1-O4	37 (24.7)	16 (21.1)	21 (28.4)
O5-O6	24 (16.0)	9 (11.8)	15 (20.3)
Major depressive disorder			
Yes	48 (31.0)	43 (55.8)	5 (6.4)
No	107 (69.0)	34 (44.2)	73 (93.6)
Continuous	*M* (*SD*)	*M* (*SD*)	*M* (*SD*)
Age	41.5 (13.7)	38.8 (11.8)	44.2 (15.0)
Intrusion ^a^	2.13 (2.14)	3.28 (2.08)	0.97 (1.46)
Avoidance ^a^	2.97 (2.57)	4.37 (2.35)	1.55 (1.95)
Negative cognitions/mood ^a^	2.67 (2.31)	4.08 (2.17)	1.24 (1.41)
Hyperarousal ^a^	2.36 (1.92)	3.46 (1.85)	1.25 (1.23)

GED, general educational development; PTSD, posttraumatic stress disorder. Except for major depressive disorder, there was no difference between those with PTSD and without PTSD in demographic characteristics.

a Range 0-10.

### 3.2 Autocorrelation and intraclass correlations

The autocorrelation, the correlation between two successive assessments collected 4 hours apart, ranged from .29 to .43 among participants with PTSD, and from .41 to .48 among those without PTSD. The ICC (the proportion of variance associated with between-person differences) among participants with and without PTSD consisted of 68.0% and 55.9% of the total variance in intrusion, 60.0% and 70.9% in avoidance, 68.1% and 73.1% in negative cognitions/mood, and 65.0% and 72.0% in the hyperarousal symptom cluster. The remaining proportion of the variances (27.0%-45.2%) were due to the within-person variance in each symptom cluster.

### 3.3 7-day day of week variation

**Figure 1:**
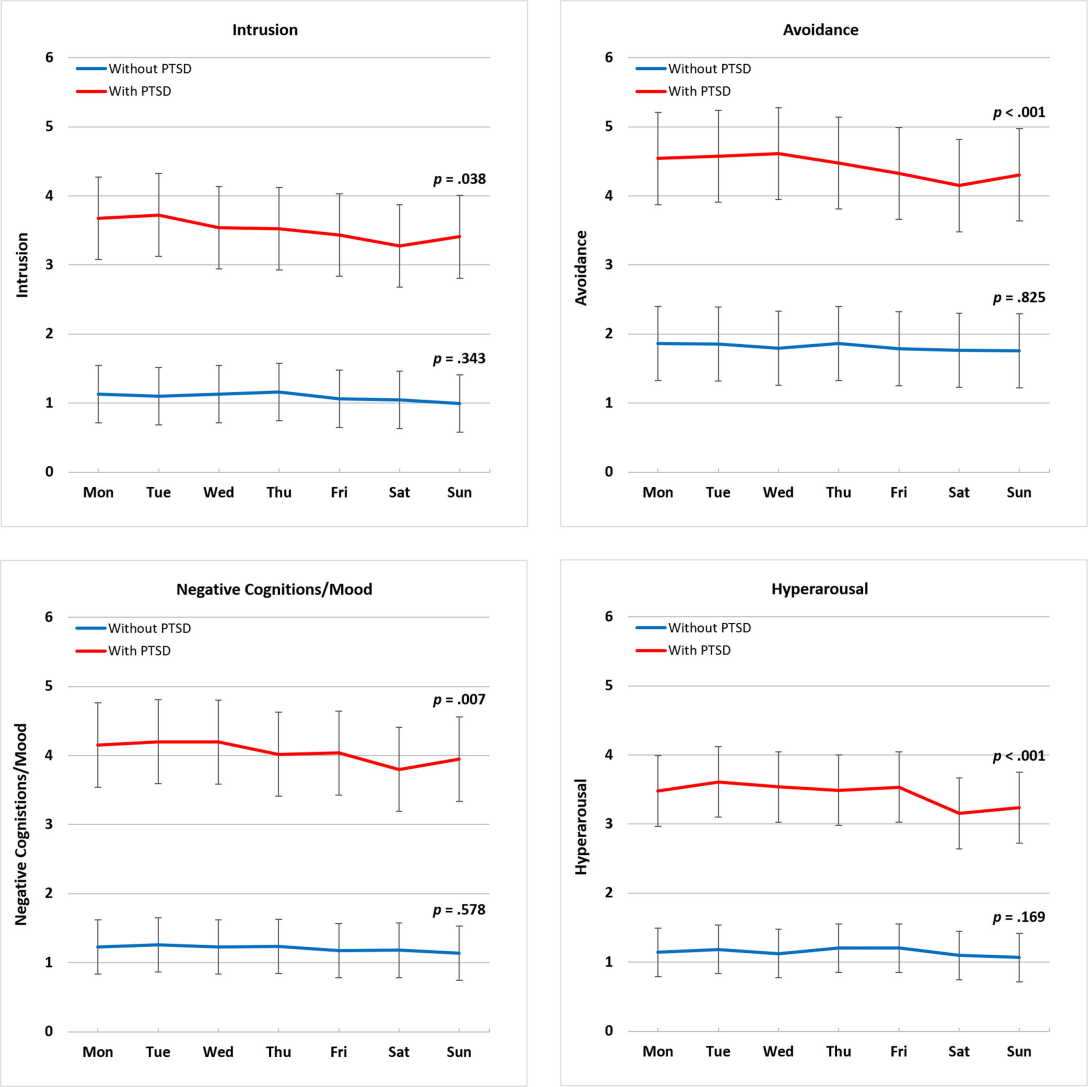
Day of week variation in PTSD symptom clusters by day of report. PTSD, posttraumatic stress disorder. Symptom cluster score range 0-10. Least squares means were obtained by estimating the four PTSD symptom clusters adjusting for demographic characteristics, including gender, age, race, and education. The *p*-value indicates whether symptoms varied significantly across the seven days of the week. Among participants with PTSD, all four clusters vary across the seven days of the week, but not among those without PTSD.

We first examined the 7-day day of week variation for each symptom cluster among participants with and without PTSD (**Figure 1**). Results of linear mixed models showed that after controlling for covariates, all symptom clusters varied across the seven days among participants with PTSD, but not among those without PTSD (intrusion with PTSD: *F*[6, 452] = 2.24, *p* = .038, without PTSD: *F*[6, 459] = 1.13, *p = .*343; avoidance with PTSD: *F*[6, 452] = 2.90, *p* = .008, without PTSD: *F*[6, 459] = 0.48, *p = .*826; negative cognitions/mood with PTSD: *F*[6, 452] = 3.00, *p* = .007, without PTSD: *F*[6, 459] = 0.79, *p = .*577; hyperarousal with PTSD: *F*[6, 451] = 5.24, *p* <.001, without PTSD: *F*[6, 459] = 1.52, *p = .*169, see **Table 2**). Further pairwise comparisons using the Tukey-Kramer method showed similar results after we controlled for multiple tests among those with PTSD.

**Table 2:** Day of week differences in PTSD symptom clusters: mixed models.

Parameter	Intrusion		Avoidance		Negative cognitions/mood		Hyperarousal	
	PTSD	Without PTSD	PTSD	Without PTSD	PTSD	Without PTSD	PTSD	Without PTSD
	Coefficient [95% CI]	Coefficient [95% CI]	Coefficient [95% CI]	Coefficient [95% CI]	Coefficient [95% CI]	Coefficient [95% CI]	Coefficient [95% CI]	Coefficient [95% CI]
Fixed effects								
Intercept	**2.79** **[1.92-3.65]**	**0.78** **[0.13-1.42]**	**4.01** **[3.04-4.98]**	**1.10** **[0.27-1.93]**	**3.91** **[3.02-4.80]**	**1.45** **[0.84-2.06]**	**2.88** **[2.13-3.63]**	**1.47** **[0.92-2.02]**
Female vs. male	-0.18 [-1.09-0.74]	0.02 [-0.69-0.73]	-0.58 [-1.61-0.49]	-0.07 [-0.99-0.85]	-0.10 [-1.05-0.86]	-0.16 [-0.84-0.52]	-0.30 [-1.11-0.50]	-0.36 [-0.96-0.24]
Age (centered at 41.5)	0.02 [-0.03-0.07]	0.01 [-0.02-0.03]	0.03 [-0.03-0.08]	0.02 [-0.01-0.05]	0.02 [-0.03-0.07]	0.01 [-0.02-0.03]	0.00 [-0.04-0.04]	0.00 [-0.02-0.02]
Non-white vs. White	0.15 [-0.84-1.15]	0.51 [-0.21-1.22]	0.07 [-1.05-1.19]	**1.16** **[0.24-2.08]**	-0.49 [-1.52-0.55]	0.03 [-0.65-0.71]	0.08 [-0.80-0.94]	-0.08 [-0.69-0.52]
Some college or lower vs. bachelor’s or higher ^a^	0.15 [-0.84-1.15]	-0.39 [-1.17-0.39]	0.63 [-0.67-1.93]	-0.53 [-1.53-0.48]	0.30 [-0.90-1.49]	-0.62 [-1.37-0.12]	0.74 [0.27-1.73]	-0.32 [-0.69-0.52]
Day of week ^b^								
Monday	**0.40** **[0.12-0.68]**	0.09 [-0.06-0.24]	**0.39** **[0.12-0.67]**	0.10 [-0.09-0.29]	**0.35** **[0.12-0.58]**	0.05 [-0.08-0.18]	**0.32** **[0.12-0.53]**	0.05 [-0.07-0.17]
Tuesday	**0.45** **[0.17-0.74]**	0.05 [-0.10-0.20]	**0.43** **[0.15-0.70]**	0.10 [-0.10-0.29]	**0.41** **[0.17-0.63]**	0.08 [-0.05-0.2]	**0.46** **[0.25-0.66]**	0.09 [-0.03-0.21]
Wednesday	0.27 [-0.01-0.55]	0.09 [-0.06-0.24]	**0.46** **[0.19-0.74]**	0.03 [-0.16-0.22]	**0.39** **[0.17-0.62]**	0.05 [-0.08-0.18]	**0.38** **[0.18-0.59]**	0.03 [-0.09-0.15]
Thursday	0.25 [-0.03-0.53]	0.11 [-0.03-0.26]	**0.33** **[0.06-0.60]**	0.10 [-0.09-0.29]	0.22 [-0.01-0.45]	0.06 [-0.07-0.18]	**0.33** **[0.13-0.54]**	0.11 [-0.01-0.22]
Friday	0.17 [-0.11-0.44]	0.02 [-0.13-0.16]	0.18 [-0.09-0.45]	0.02 [-0.16-0.21]	**0.24** **[0.01-0.46]**	-0.01 [-0.13-0.12]	**0.38** **[0.18-0.58]**	0.11 [-0.01-0.22]
Sunday	0.14 [-0.15-0.42]	-0.05 [-0.20-0.10]	0.16 [-0.12-0.43]	-0.01 [-0.20-0.18]	0.15 [-0.08-0.38]	-0.04 [-0.17-0.09]	0.09 [-0.12-0.29]	-0.03 [-0.15-0.09]
Random effects								
Between-person variance	**4.03**	**1.95**	**5.20**	**3.25**	**4.44**	**1.78**	**3.10**	**1.40**
Autocorrelation	**0.41**	**0.41**	**0.29**	**0.45**	**0.43**	**0.45**	**0.37**	**0.48**
Within-person variance	**3.28**	**0.96**	**3.55**	**1.50**	**2.10**	**0.70**	**1.75**	**0.56**
Intraclass correlation ^c^	0.55	0.67	0.59	0.68	0.68	0.72	0.64	0.71

PTSD, posttraumatic stress disorder. PTSD group *n* = 80, Without PTSD group *n* = 79.

^a^Bachelor’s degree or higher was set as the reference. ^b^Saturday was set as the reference. Results of linear mixed models showed that after controlling for covariates, all symptom clusters varied across the seven days among participants with PTSD, but not among those without PTSD (intrusion with PTSD: *F*[6, 452] = 2.24, *p* = .038, without PTSD: *F*[6, 459] = 1.13, *p* = .343; avoidance with PTSD: *F*[6, 452] = 2.90, *p* = .008, without PTSD: *F*[6, 459] = 0.48, *p* = .826; negative cognitions/mood with PTSD: *F*[6, 452] = 3.00, *p* = .007, without PTSD: *F*[6, 459] = 0.79, *p* = .577; hyperarousal with PTSD: *F*[6, 451] = 5.24, *p* <.001, without PTSD: *F*[6, 459] = 1.52, *p* = .169). ^c^Intraclass Correlation was calculated by the ratio of between-person variance and the total variance.

Bold values indicate significance at p < .05.

### 3.4 Weekday versus weekend differences

**Figure 2:**
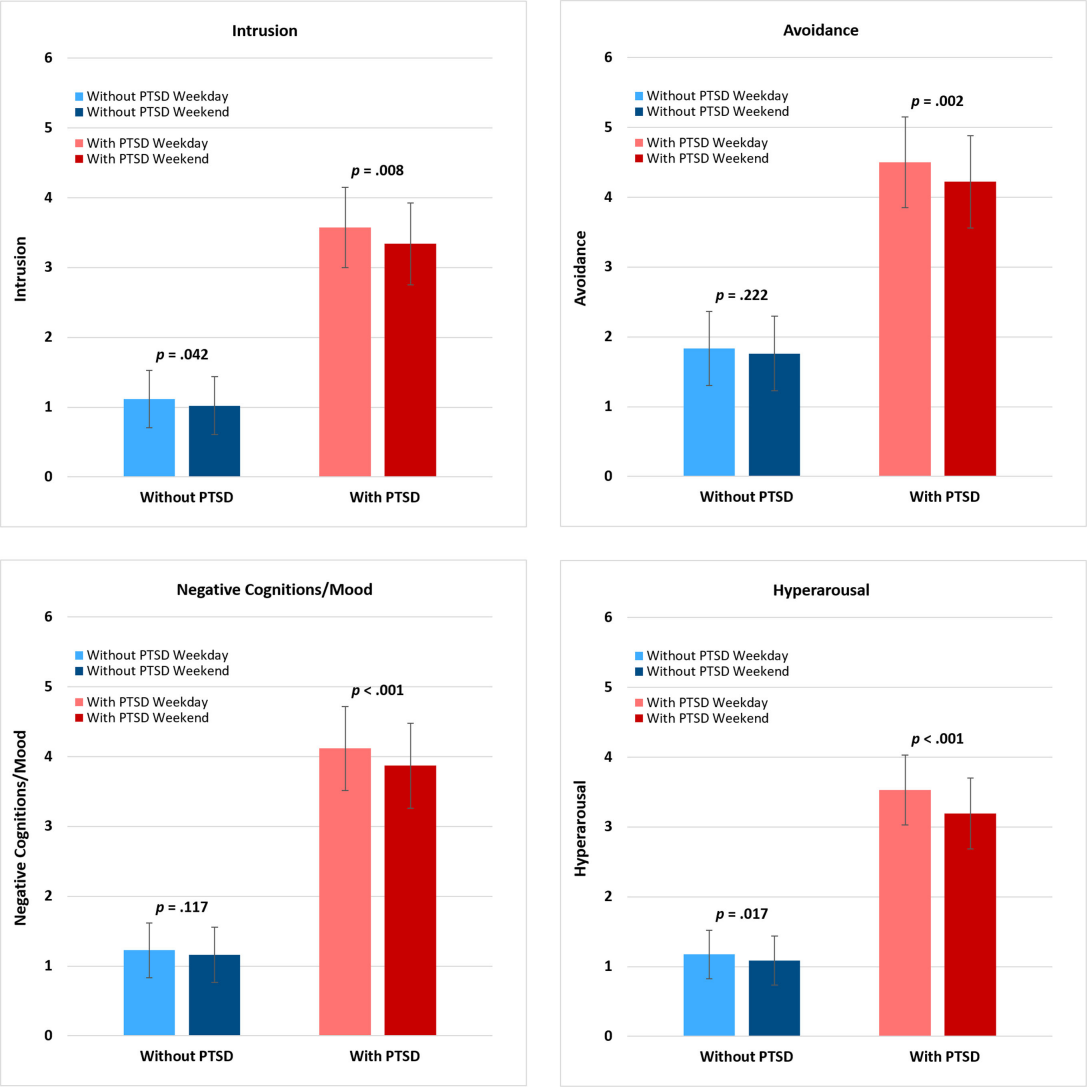
Weekday/weekend variation in PTSD symptom clusters by day of report. PTSD, posttraumatic stress disorder. Symptom cluster score range 0-10. Least squares means were obtained by estimating the four PTSD symptom clusters adjusting for demographic characteristics, including gender, age, race, and education. The *p*-value indicates whether symptoms varied significantly between weekdays and weekends. Among participants with PTSD, all four clusters were different between weekdays and weekends; among participants without PTSD, the intrusion and hyperarousal symptom clusters were different between weekdays and weekends.

We then examined differences in the four symptom clusters between weekdays and weekends among participants with and without PTSD (**Figure 2**). Among participants with PTSD, all four symptom clusters were higher on weekdays than weekends (intrusion: *p* = .008, avoidance: *p* = .002, negative cognitions/mood: *p* <.001, and hyperarousal: *p* <.001); among those without PTSD, there was a weekday versus weekend difference only for intrusion (*p* = .042) and hyperarousal (*p* = .017) symptom clusters.

Among participants with PTSD, the weekday and weekend differences were attenuated but remained significant or marginally significant when random slopes for weekday and weekend differences were included, (intrusion: *p* = .056, avoidance: *p* = .031, negative cognitions: *p* = .012, and hyperarousal: *p* <.001).

## 4 Discussion

Using an EMA approach, this study examined the day of the week variation in each of the DSM-5 PTSD symptom clusters (intrusion, avoidance, negative cognitions/mood, and hyperarousal) across 15 days in individuals with and without PTSD. All four symptom clusters varied across the seven days of the week among individuals with PTSD, but not among individuals without PTSD (see **Figure 1**). The symptom clusters all showed a similar pattern; symptoms were highest on weekdays and declined to the lowest point on Saturday/Sunday. These results are consistent with our previous findings that showed the same pattern in the overall PTSS sum score (10) and suggest there are at least some common drivers for the four symptom clusters since they all changed in a similar way.

We also compared weekdays (Monday through Friday) versus weekends (Saturday and Sunday) to focus on potential differences between traditional work and non-work days. Among those with PTSD, all four PTSD symptom clusters were higher on weekdays than weekends. In this analysis, of interest is that among those without PTSD the intrusion and hyperarousal clusters were higher on weekdays than weekends, but there was no weekday/weekend difference in the avoidance and negative cognitions/mood clusters (see **Figure 2**). These differences in the non-PTSD group may reflect that these symptom clusters capture daily stress experiences as well as traumatic stress responses. Whether the daily stress and traumatic stress are the same neurobiological mechanisms or how they are similar and different (e.g., overlapping) would be important to consider. The absence of weekday versus weekend differences in avoidance and negative cognitions/mood may similarly reflect that these clusters are not a significant part of normal daily stressor responses but reflect more pathological/disorder-based symptoms with unique neurobiology associated with PTSD only.

Our findings of daily variation in PTSD symptom clusters highlight the need to develop more person-specific interventions. Recent studies have examined whether or not treatments given at different times influence outcomes (41–43). It may be possible that there are optimal times for giving psychiatric medication or psychotherapeutic treatment. Prior studies have identified hyperarousal as a leading predictor of subsequent symptom severity in the other PTSD symptom clusters (17, 18). Targeting the delivery of interventions for hyperarousal symptoms to specific days of the week when hyperarousal symptoms are highest may lead to better outcomes among all symptom clusters. Studies examining the contemporary and temporal relationships of PTSD symptoms may be helpful in pinpointing specific symptoms to target for intervention and timepoints for altering treatment approaches to obtain maximum treatment efficacy (44, 45). Alternatively, individuals may be more receptive to the most challenging aspects of psychotherapy on days when their symptom levels are lowest, thereby increasing the saliency of the intervention (e.g., prolonged exposure) and reducing the adversity that leads to treatment dropout. In addition, cognizance of day-to-day variation in PTSD symptom clusters may be particularly important for massed PTSD treatment programs that include daily intensive psychotherapy over short (e.g., 2 week) periods of time (46).

Daily variation in PTSD symptom clusters may be related to a number of different factors. Studies have shown that sleep disturbances affect PTSS (25, 47, 48). Sleep duration varies across the days of the week for individuals with and without PTSD, and those with PTSD have more trouble falling asleep and lower sleep quality on weekdays compared to weekends (25). Having a shorter sleep duration and more trouble falling asleep the previous night predicts higher PTSS the following day among individuals with PTSD (24). In addition, nightmares predict each of the four PTSD symptom clusters (49) and short sleep duration is associated with a subsequent increase in intrusion symptoms (50, 51). There may be day-to-day differences in factors that trigger PTSD symptoms. In a military population, exposures to uniforms, equipment, weapons, training exercises, injured soldiers, or combat-related stimuli, which may be present in the work day environment but not in the non-work day environment, may trigger PTSD symptoms. There may also be psychosocial and lifestyle factors such as exercise (52), leisure activities (53), and social interactions (54) that differ between weekdays and weekends and are associated with improved mood, greater social support, and reductions in arousal that buffer against PTSD symptoms. The combination of potentially contributing neurobiological, environmental, psychosocial, and lifestyle factors experienced by the individual are likely to culminate in the ebb and flow of PTSD symptoms observed from day to day. To further examine these factors, studies are needed that capture daily activities and evaluate their temporal association to variation in PTSD symptom clusters. Identifying precipitating and potentially protective factors may suggest interventions to reduce PTSD symptoms. Future studies may wish to explore traumatic event reminders, interpersonal conflicts, and stressful events in both the work and home environments. Future studies may also examine the extent to which repeated self-monitoring influences PTSD symptom levels and whether assessment items themselves or the timing, frequency, and duration of responding enhances symptom change.

The present study has several limitations. First, measures of PTSS, trauma exposure, and PTSD were obtained by self-report. For PTSS, this limitation is partially mitigated by collection of daily symptom assessments at the time of the behavior of interest or within hours afterwards. The measures for trauma exposure and PTSD had the potential for retrospective recall bias and may be less accurate compared to a clinical psychiatric assessment. Although, all assessments included well-validated self-assessment measures. Second, since we did not track participants’ daily activities, such as work schedules, we do not know to what extent the PTSD symptom clusters vary with daily activities. Third, the low level of symptom severity among participants without PTSD may have introduced a floor effect, potentially contributing to the group differences between participants with and without PTSD (55). Fourth, because our focus was on exploring cluster-specific patterns in day of week variation, we did not model the full symptom network. Future work utilizing multivariate models would be well-suited to capturing interdependencies among clusters. Fifth, given the exploratory nature of the current study, we did not include potential predictors that might explain the observed day of week differences. Future research is recommended to investigate underlying mechanisms such as sleep disturbances that may be associated with both day of week and PTSD symptom clusters. Sixth, future studies are needed to explore intra-individual variability not accounted for by day of week variation, as indicated by the wide confidence intervals around the least squares means in the current study. Finally, subjects were recruited from a military medical center, which may limit the generalizability of our findings.

This study provides further evidence that PTSD symptom clusters do not remain static; among individuals with PTSD, all four symptom clusters vary across the seven days of the week and differ between weekdays and weekends. Individuals without PTSD also showed weekday/weekend differences but only in intrusion and hyperarousal suggesting these symptoms are partly related to everyday versus traumatic stress. Further study of PTSD symptom cluster variation may lead to a better understanding of how symptoms change over time and the neurobiological, environmental, psychosocial, and lifestyle factors associated with change. Identification of the factors associated with day-to-day variation in PTSD symptom clusters may be important for understanding the neurobiology of PTSD and aid in the development of timely and effective interventions to treat PTSD.

## Data Availability

The raw data supporting the conclusions of this article will be made available by the authors, without undue reservation.
