# Supplementary material for: Implications for optimizing treatment timing: day of week variation in PTSD symptom clusters
Source: Front Psychiatry. 2025 Oct 17;16:1599424. doi: 10.3389/fpsyt.2025.1599424 (PMC12576295; doi:10.3389/fpsyt.2025.1599424)
Supplement: Supplementary file 1 [file Supplementaryfile1.docx]

**Supplementary Material**

**Figure S1**

Eighteen Posttraumatic Stress Symptoms and Posttraumatic Stress Disorder Symptom Clusters

Not At All Moderately Extremely

|  | | |
| --- | --- | --- |
| Intrusion | 1. Repeated, disturbing, and unwanted memories of the stressful experience? | 0 1 2 3 4 5 6 7 8 9 10 |
|  | 3. Suddenly feeling or acting as if the stressful experience were actually happening again (as if you were actually back there reliving it)? | 0 1 2 3 4 5 6 7 8 9 10 |
|  | 4. Feeling very upset when something reminded you of the stressful experience? | 0 1 2 3 4 5 6 7 8 9 10 |
|  | 5. Having strong physical reactions when something reminded you of the stressful experience (for example, heart pounding, trouble breathing, sweating)? | 0 1 2 3 4 5 6 7 8 9 10 |
| Avoidance | 6. Avoiding memories, thoughts, or feelings related to the stressful experience? | 0 1 2 3 4 5 6 7 8 9 10 |
|  | 7. Avoiding external reminders of the stressful experience (for example, people, places, conversations, activities, objects, or situations)? | 0 1 2 3 4 5 6 7 8 9 10 |
| Negative Cognitions/Mood | 8. Trouble remembering important parts of the stressful experience? | 0 1 2 3 4 5 6 7 8 9 10 |
|  | 9. Having strong negative beliefs about yourself, other people, or the world (for example, having thoughts such as: I am bad, there is something seriously wrong with me, no one can be trusted, the world is completely dangerous)? | 0 1 2 3 4 5 6 7 8 9 10 |
|  | 10. Blaming yourself or someone else for the stressful experience or what happened after it? | 0 1 2 3 4 5 6 7 8 9 10 |
|  | 11. Having strong negative feelings such as fear, horror, anger, guilt, or shame? | 0 1 2 3 4 5 6 7 8 9 10 |
|  | 12. Loss of interest in activities that you used to enjoy? | 0 1 2 3 4 5 6 7 8 9 10 |
|  | 13. Feeling distant or cut off from other people? | 0 1 2 3 4 5 6 7 8 9 10 |
|  | 14. Trouble experiencing positive feelings (for example, being unable to feel happiness or have loving feelings for people close to you)? | 0 1 2 3 4 5 6 7 8 9 10 |
| Hyperarousal | 15. Irritable behavior, angry outbursts, or acting aggressively? | 0 1 2 3 4 5 6 7 8 9 10 |
|  | 16. Taking too many risks or doing things that could cause you harm? | 0 1 2 3 4 5 6 7 8 9 10 |
|  | 17. Being “superalert” or watchful or on guard? | 0 1 2 3 4 5 6 7 8 9 10 |
|  | 18. Feeling jumpy or easily startled? | 0 1 2 3 4 5 6 7 8 9 10 |
|  | 19. Having difficulty concentrating? | 0 1 2 3 4 5 6 7 8 9 10 |

*Note*. Two items from the Posttraumatic Stress Disorder Checklist for DSM-5 (PCL-5) were sleep-related (i.e., *2. Repeated, disturbing dreams of the stressful experience* and *20. Trouble falling or staying asleep*) and were not appropriate for use on all daily assessments.
